# Habitat Suitability Assessment of Key Wildlife in Hainan Tropical Rainforest Based on ESDM

**DOI:** 10.3390/life15020323

**Published:** 2025-02-19

**Authors:** Wutao Yao, Jin Yang, Yong Ma, Lixi Liu, Erping Shang, Shuyan Zhang

**Affiliations:** 1 Key Laboratory of Earth Observation of Hainan Province, Hainan Aerospace Information Research Institute, Sanya 572029, China; yaowt@aircas.ac.cn (W.Y.); yangjin@aircas.ac.cn (J.Y.); llxsdyp@bjfu.edu.cn (L.L.); shangep@aircas.ac.cn (E.S.); zhangshuyan@aircas.ac.cn (S.Z.); 2 Aerospace Information Research Institute, Chinese Academy of Sciences, Beijing 100094, China

**Keywords:** Hainan tropical rainforest, ESDM, habitat suitability, Jianfengling

## Abstract

Hainan tropical rainforest is the largest contiguous tropical rainforest in China, but it has experienced increasing disturbances from anthropogenic activities in recent decades due to economic and social development. However, the current status of wildlife habitats within the rainforest remains insufficiently studied, lacking systematic and scientific assessments necessary to guide effective biodiversity conservation strategies. This study focuses on Jianfengling area of Hainan tropical rainforest, using wildlife infrared camera monitoring data and habitat environmental factor data collected through multi-source monitoring in 2020–2021. By applying the Ensemble Species Distribution Model (ESDM), we assessed the spatial distribution of habitat suitability and its influencing factors for seven representative wildlife species, as well as the overall spatial distribution of multi-species habitat suitability. The results indicate that wildlife habitat suitability in Jianfengling study area exhibits a spatial pattern of high suitability in the central regions and low suitability in surrounding areas. Anthropogenic activities and DEM were identified as the most significant factors influencing habitat selection, with most species favoring mid and high altitude areas (500–1000 m) where human activities are less prevalent. This study provides scientific support for tropical rainforest management authorities to optimize resource allocation, develop dynamic monitoring strategies, and implement effective conservation measures.

## 1. Introduction

Habitat serves as the foundation and home for wildlife, with its quality, distribution, and conservation status directly influencing ecosystem stability and biodiversity preservation [1]. Tropical rainforests, among the most complex and diverse ecosystems on Earth, play an irreplaceable role in global climate regulation, carbon storage, and the water cycle [2]. They also harbor numerous unique plant and animal species, contributing significantly to global biodiversity [3]. However, in recent years, tropical rainforests have faced unprecedented biodiversity threats due to intensified human activities, such as deforestation, agricultural expansion, and infrastructure development. These activities have led to habitat fragmentation, degradation, and loss, severely impacting the species that depend on these ecosystems [4,5]. Consequently, comprehensive monitoring and assessment of tropical rainforest wildlife habitats are essential for the scientific development of effective conservation strategies and measures [6].

Traditional wildlife habitat monitoring and assessment typically relies on ground surveys or the sample line method to collect wildlife location data [7]. These data are then combined with long-term behavioral observations to map rough habitat boundaries. However, advancements in satellite and sensor technology have introduced more efficient tools for monitoring and assessing wildlife habitats. Satellite remote sensing technology, with its ability to capture large-scale, wide-area, and long-term data, accurately tracks changes in vegetation cover, land use dynamics, habitat degradation, and the impact of human activities [6]. By integrating these data with species distribution models (SDM), researchers can achieve a relatively high level of accuracy in analyzing and assessing wildlife habitat conditions [8]. SDM enable precise mapping of suitable and core habitats for various species [9]. For example, Fotang et al. [10] used maximum entropy (MaxEnt) to map the suitable habitats of chimpanzees in the Kom-Wum Forest Reserve in the northwest of Cameroon, and it was clearly identified that the suitable chimpanzee habitats within the reserve are shrinking and degrading. Paniw et al. [11] proposed a multi-species habitat modeling framework based on interspecific interactions and optimized by Bayesian, which provided theoretical support for understanding the dynamics of species coexistence. The research by Rahmadiyanti et al. [12] showed that the expansion of oil palm plantations has severely squeezed the living space of wild animals, and climate change is altering the pattern of habitat suitability, and some species may face the risk of extinction due to a lack of adaptability. These studies collectively provide critical scientific support for wildlife conservation and habitat management. However, SDM have several limitations, particularly regarding their applicability. When different SDMs are applied across distinct ecological regions and species, significant variations can occur, leading to unreliable predictions and assessments from any single model in comprehensive studies. To solve these issues, ensemble species distribution model (ESDM) has been proposed. ESDM can improve prediction accuracy, generalizability, and robustness by integrating the results of multiple models. Currently, ensemble methods are widely used in habitat suitability prediction and assessment research [13,14].

Hainan Tropical Rainforest is the largest, most concentrated, diverse, and well-preserved contiguous continental island-type tropical rainforest in China. Its biodiversity index is comparable to that of the Amazon rainforest, a global biodiversity hotspot, and it serves as the only habitat for the critically endangered Hainan gibbon (*Nomascus hainanus*) [15]. However, Hainan’s tropical rainforests are increasingly affected by economic development and urbanization, leading to heightened anthropogenic interference and deteriorating ecological conditions [16]. To address these challenges, China established Hainan Tropical Rainforest National Park in 2021 by merging several rainforest reserves in Hainan Province [17]. The park aims to protect tropical rainforest biodiversity and enhance the ecological services provided by these rainforests [18]. Despite these efforts, the high temperature and humidity, along with the complexity and variability of the rainforest environment, have resulted in a significant lack of historical monitoring data. Additionally, systematic and in-depth research on species habitat distribution, the factors influencing habitat suitability, and multi-species habitat assessment remains scarce. This has led to unclear functional zoning within the national park, conflicting and blurred boundaries, and challenges in planning and implementing effective conservation measures. These issues remain critical barriers to the development of national parks and the execution of conservation strategies.

This study comprehensively utilized wildlife data from infrared camera monitoring in Jianfengling area of Hainan Tropical Rainforest and habitat environmental factor data derived from multi-source remote sensing. The Ensemble Species Distribution Model (ESDM) was applied to analyze the distribution of suitable habitats for seven key wildlife species in the region. The study also examined the primary factors influencing habitat selection and their respective modes of impact. Furthermore, conservation status and endangerment levels of the species were incorporated to assess the spatial distribution of suitable habitats for multiple species. This analysis provides a scientific foundation for optimizing resource allocation, formulating dynamic management strategies, and implementing effective conservation efforts within national parks.

## 2. Materials and Methods

### 2.1. Study Area

Jianfengling area of Hainan Tropical Rainforest National Park, formerly known as Hainan Jianfengling National Nature Reserve, is situated in the southwestern region of Hainan Island, spanning Ledong Country and Dongfang City. Its geographical coordinates range from 108°44′–109°02′ E longitude and 18°35′–18°52′ N latitude [19]. In 1960, this area is classified as a forest ecosystem nature reserve and it is the first nature reserve in Hainan Province. It was designated as a national nature reserve on 1 August 2002. On 12 October 2021, it was officially merged with several other protected areas in Hainan to form Hainan Tropical Rainforest National Park [20].

Jianfengling area is a significant component of Hainan Tropical Rainforest National Park. It represents the tropical rainforest with the lowest latitude, most intact vertical structure, largest area, and best preservation in China. The region is characterized by extensive tropical semi-deciduous monsoon rainforests, tropical evergreen monsoon rainforests, and tropical montane rainforests. Jianfengling area is predominantly mountainous, with its highest peak, Mount Jianfengling, rising to an elevation of 1412.5 m. The climate is tropical monsoonal, with year-round warm temperatures and distinct wet and dry seasons. Summers are hot and humid, with abundant rainfall, while winters are dry and cool, with minimal precipitation. The mean annual temperature is 24.5 °C, and the average annual precipitation is 2265.8 mm [21]. As one of the most biologically diverse regions in the country, Jianfengling is often referred to as the “gene pool of species at the northern edge of the tropics”. Jianfengling is home to over 371 documented vertebrate species, including a notable concentration of rare and endangered species, such as the Hainan peacock-pheasant (*Polyplectron katsumatae*), Hainan partridge (*Arborophila ardens*), Chinese pangolin (*Manis pentadactyla*), and clouded leopard (*Neofelis nebulosa*) [22].

The biodiversity of Jianfengling study area has faced significant threats in recent decades due to population growth, agricultural expansion, and the development of tourism in Hainan. Consequently, conducting monitoring and assessment studies of wildlife and their habitats in this region is of critical ecological importance.

### 2.2. Species Distribution Data

**Figure 1:**
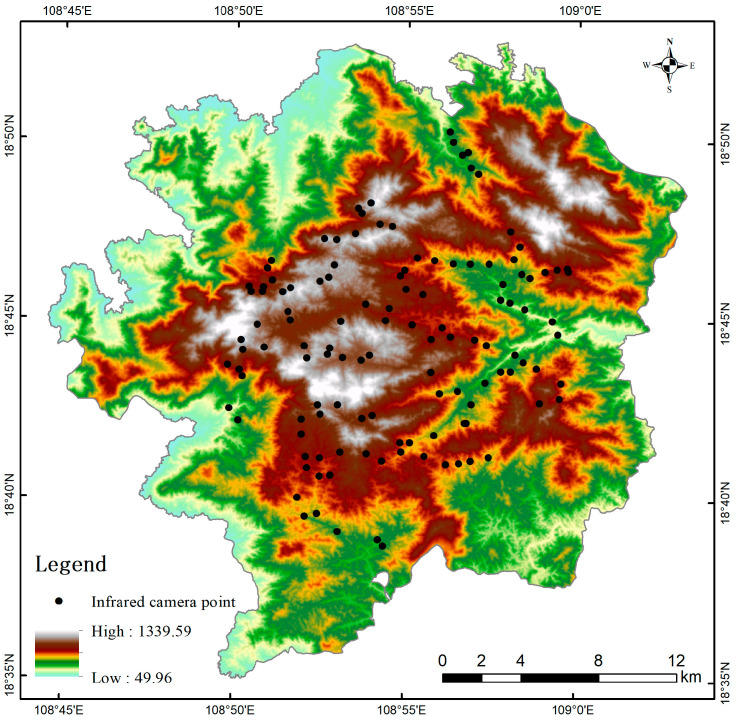
Elevation distribution and infrared camera monitoring points in the study area.

Infrared cameras were deployed in the study area from October 2020 to November 2021 (Figure 1), capturing a total of 151,192 images and videos over approximately 14 months. After processing, 9414 independent and valid photographs were obtained, documenting 17 species of wild mammals. Based on factors such as endangerment status, ecological niche differentiation, and relative abundance index (RAI), seven species were selected for subsequent analyses: rhesus macaque (*Macaca mulatta*), common palm civet (*Paradoxurus hermaphroditus*), Hainan muntjac (*Muntiacus nigripes*), ferret badger (*Melogale moschata*), red-legged long-nosed squirrel (*Dremomys pyrrhomerus*), wild boar (*Sus scrofa*), and Asiatic brush-tailed porcupine (*Atherurus macrourus*). Among the seven species, *Macaca mulatta*, *Paradoxurus hermaphroditus*, and *Muntiacus nigripes* are listed as State Class II protected animals, with *Muntiacus nigripes* being endemic to Hainan [23]. *Melogale moschata* and *Dremomys pyrrhomerus* are categorized as Near Threatened on the Red List of Chinese Vertebrates [24]. In contrast, the *Sus scrofa* and *Atherurus macrourus* are widely distributed mammals. Detailed information about these species is provided in Table 1 and Table 2.

### 2.3. Environmental Factor Data

To evaluate the habitat suitability of the seven species in this study, various environmental factors were extracted from remote sensing data, meteorological records, and other multi-source heterogeneous datasets. The specific details are as follows:

#### 2.3.1. Vegetation Factor

Although Normalized Difference Vegetation Index (NDVI) is one of the most commonly used remote sensing vegetation indices, the Enhanced Vegetation Index (EVI) was chosen to represent vegetation characteristics in the study area due to NDVI’s saturation in densely vegetated regions like tropical rainforests [25]. Using Landsat-8 satellite data, semi-monthly EVI products were generated for the period from October 2020 to November 2021. These datasets were subsequently de-clouded and synthesized into annual averages, resulting in final EVI products with a spatial resolution of 30 m.

#### 2.3.2. Topographical Factors

Topographic factors included elevation, slope, and aspect. Elevation data were obtained from the ASTER GDEM v3 dataset, sourced from the Geospatial Data Cloud, with a spatial resolution of 30 m [26]. Slope and aspect were derived using the spatial processing tools in ArcGIS, based on the Digital Elevation Model (DEM) data.

#### 2.3.3. Land Use/Cover Factor

The dataset is based on the 2020 multi-period land use/cover remote sensing monitoring product developed by the Institute of Geographic Sciences and Natural Resources Research, Chinese Academy of Sciences. This product classifies land features into 6 main categories and 25 subcategories according to their utilization attributes and intrinsic characteristics [27]. It is derived from Landsat satellite data with a spatial resolution of 30 m and is compatible with EVI data for analysis.

#### 2.3.4. Meteorological Factors

The meteorological data are sourced from the WorldClim version 2.1 dataset, which has a spatial resolution of 30 arc-seconds and includes 19 bioclimatic variables, such as mean temperature, minimum and maximum temperatures, and precipitation. This dataset is widely used in GIS and spatial modeling analyses [28].

#### 2.3.5. Anthropogenic Activity Factors

Road data from OpenStreetMap (OSM) 2020 were obtained, and detailed road information was extracted from a high-resolution remote sensing image (Google Earth map, 0.5 m) of the study area [29,30]. These data were then integrated to create the road vector data for the study area. The spatial distance calculation tool in ArcGIS was used to derive the distance from each point in the research area to the nearest road. This result was ultimately made into a raster layer with a resolution of 30 m, serving as an indicator of the influence of anthropogenic activities.

After generating data products for each habitat factor category, all data were resampled to a spatial resolution of 30 m. Variables with a variance inflation factor (VIF) exceeding 10 or a Pearson correlation coefficient of 0.7 or higher were removed using the USDM package in R 4.1.1 and the Seaborn library in Python 3.9 [31,32,33].

### 2.4. Species Distribution Modeling and Accuracy Evaluation

In this study, the ESDM was applied to assess habitat suitability for individual species. The ESDM was selected because extensive research has shown that it outperforms single SDM in terms of prediction accuracy. Moreover, the ESDM offers improved generalization capabilities and robustness [34]. Three models—MaxEnt, random forest (RF), and support vector machine (SVM)—were integrated for ensemble learning, as these models are widely used and have demonstrated superior predictive performance in habitat suitability studies [35].

The SSDM package (version 0.2.9) was utilized to construct the ESDM within the R 4.3.3 environment. Only SDMs with an area under the curve (AUC) value exceeding 0.85 were included in the ensemble model. The weight of each SDM was determined by the ratio of its AUC value to the sum of the AUC values of all integrated models. The weights were calculated using the following formula [35]:

(1) $$W_{i}=\frac{r_{i}}{\sum_{j=1}^{h}r_{j}}$$

In this context, $W_{i}$ represents the weight assigned to the ith model, $r_{i}$ denotes the AUC value of the ith model, and h is the total number of single models with AUC values greater than 0.85.

The species distribution data includes only species presence records; however, developing a robust predictive model requires the inclusion of species absence data. Therefore, the random selection method in the SSDM package was used to generate absence data, matching the number of presence records for all species in the study area.

The ESDM divides species distribution data into two subsets: a training set (80%) and a test set (20%). This process is repeated 10 times using the bootstrap test method. In the bootstrap test, replacement sampling is performed to create training datasets of the same size as the original dataset. To minimize the influence of the number of runs, the repetition count was set to 20. Model predictions were output as raster data, with pixel values ranging from 0 to 1, representing a gradient of increasing habitat suitability. The contribution of environmental factors to the model was quantified using the default jackknife method in the SSDM package, which also assessed the environmental preferences of different species.

To evaluate the predictive performance of the model, two key metrics were employed: the AUC and the true skill statistic (TSS) [36,37]. The AUC ranges from 0.5 to 1, where values closer to 1 indicate superior predictive performance, while a value of 0.5 suggests the model performs no better than random guessing. The TSS ranges from −1 to 1, with 1 indicating optimal prediction accuracy, 0 signifying performance equivalent to random guessing, and values below 0 denoting performance worse than random guessing.

### 2.5. Multi-Species Habitat Suitability Assessment

In this study, a scoring system was employed to ascertain the conservation value corresponding to different species. The calculation criterion is based on three aspects: the conservation status, endangerment and trade restrictions of the species selected for this study [38,39]. The conservation value of the species was determined by calculating the mean value of the three aspects, as follows:

(2) $$I=\frac{I_{grade}+I_{endangered}+I_{CITES}}{3}$$

The grade of species conservation is represented by the letter “*I_grade_*”; the grade of endangered species is represented by the letter “*I_endangered_*”; and the level of trade control is represented by the letter “*I_CITES_*”. The combined evaluation result is represented by the letter “*I*”, which is the average value of the aforementioned letters. Table 3 illustrates the scores assigned to the various grades of the three evaluation modules employed in this study.

Once the conservation value of the various species had been determined, the ratio of the conservation value of each species to the sum of the conservation value of the seven species was calculated. This ratio was then used as the weighting factor in the multi-species habitat suitability assessment of the species. Ultimately, the results of the spatial distribution of multi-species habitat suitability were obtained through weighted superposition [38,39]. The following formula is used to calculate the weight of single-species habitat suitability assessment results:

(3) $$P_{i}=\frac{I_{i}}{\sum_{j=1}^{7}I_{j}}$$

In the calculation of a multi-species habitat suitability distribution, the weight of the ith species habitat suitability distribution result is denoted by $P_{i}$. The conservation value of the ith species is denoted by $I_{i}$.

## 3. Results

### 3.1. Analysis of Model Prediction Performance and Contribution of Environmental Factors

To evaluate the predictive performance of the model, two key metrics were utilized. The accuracy of the ESDM in predicting suitable habitats for the seven species was assessed, with detailed results provided in Table 4. The AUC values exceeded 0.85, reaching a maximum of 0.93, while the TSS values ranged from 0.482 to 0.648. Considering that TSS values are affected by the accuracy of randomly generated absence data, it can be concluded that the model’s predictions are reliable.

**Figure 2:**
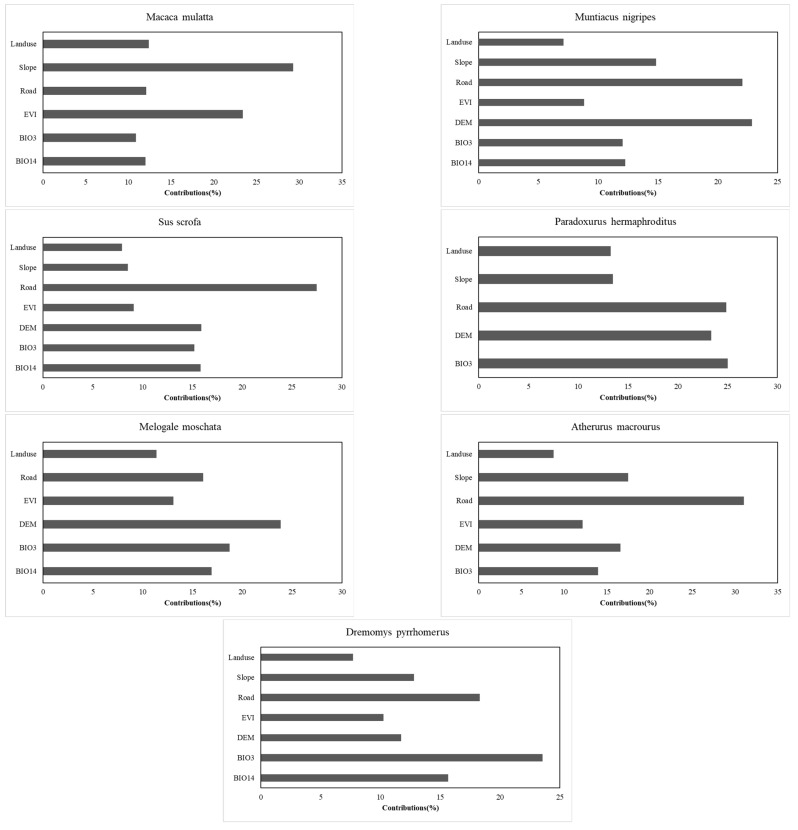
Contributions of environmental factors to the results of habitat suitability prediction for 7 species.

Figure 2 depicts the contributions of environmental factors to the habitat suitability assessments for the seven species. After screening, seven key environmental factors were identified (Table 5): Land use/cover (Landuse), distance to roads (Road), EVI, DEM, slope, isothermality (BIO3), and precipitation of the driest month (BIO14). For *Macaca mulatta*, slope was the most significant factor, contributing approximately 30%, followed by EVI (23.4%). For *Muntiacus nigripes*, DEM (22.9%) and distance to roads (22.1%) were the most important factors, with slope ranking third at 14.9%. Distance to roads was the primary factor for *Sus scrofa*, contributing 27.5%. For the *Paradoxurus hermaphroditus*, distance to roads, DEM, and BIO3 each contributed between 23% and 25%, while slope and land use contributed approximately 13%. In the case of *Melogale moschata*, DEM had the highest contribution (23.8%), with other factors contributing between 11% and 18%. For *Atherurus macrourus*, distance to roads was the most influential factor (31%), surpassing slope, which ranked second at 17.5%. Finally, for *Dremomys pyrrhomerus*, BIO3 was the most significant factor (23.6%), followed by distance to roads (18.3%) and BIO14 (15.7%).

### 3.2. Spatial Distribution of Habitat Suitability for Single Species

**Figure 3:**
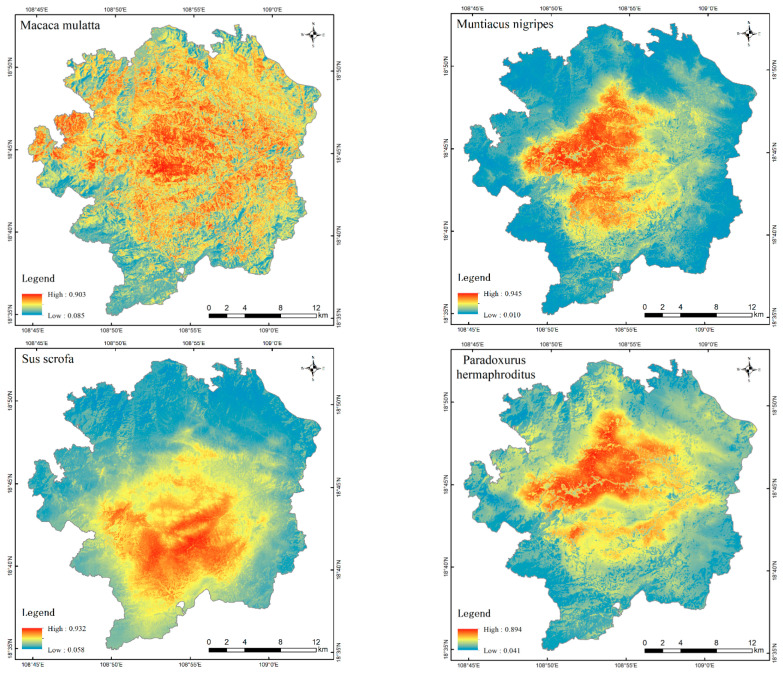

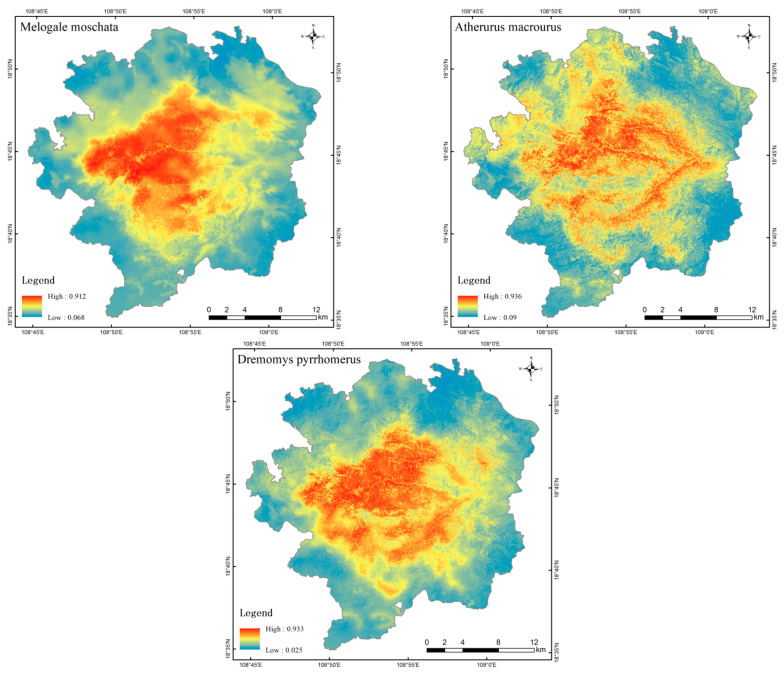
Spatial distribution of habitat suitability for single species.

The results of the habitat suitability distribution assessment for each species, conducted using the ESDM, are presented in Figure 3. The color gradient from blue to red indicates the gradual increase in habitat suitability for each species. The distribution of highly suitable habitats for *Macaca mulatta* was more extensive than that for the other six species, with the exception of a small area in the south-west of the study area, which had relatively low suitability. In contrast, all other areas of the study area exhibited a distribution of highly suitable habitats. In particular, the central part of the study area, which is characterized by extensive red patches, exhibits the highest habitat suitability. The area deemed most suitable for *Sus scrofa* was concentrated in the southern sector of the study area. With the exception of *Macaca mulatta* and *Sus scrofa*, the highly suitable habitats of the remaining five species were primarily concentrated in the central region of the study area, with significantly lower habitat suitability observed in the area surrounding the outer boundary of the study area. The distribution of highly suitable habitats for *Muntiacus nigripes* and *Melogale moschata* exhibited significant overlap, with the highest concentration observed in the west-central region of the study area. The distribution of suitable habitat for *Paradoxurus hermaphroditus* and *Atherurus macrourus* was more proximate and mainly distributed in two regions, with the big part in the north-central of the study area and the small part in the south-central of the study area. In contrast, the distribution of suitable habitat for *Dremomys pyrrhomerus* were more evenly distributed in the central part of the study area. In general, the central core of the study area can be considered a relatively high-suitability area for all species.

### 3.3. The Spatial Distribution of Multi-Species Habitat Suitability

**Figure 4:**
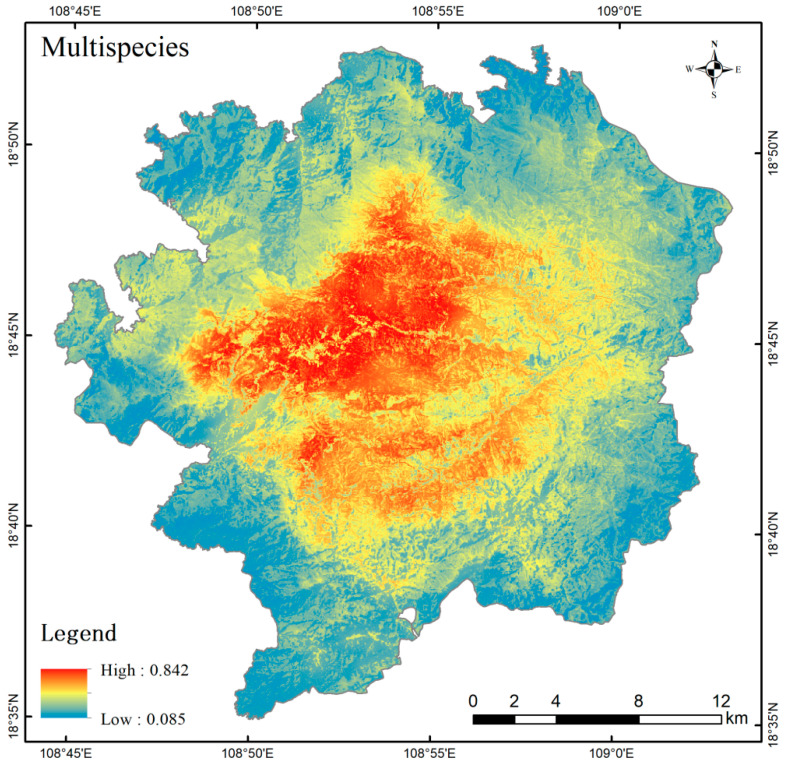
Spatial distribution of multi-species habitat suitability.

The conservation value scores of the seven species were calculated based on three factors: conservation status, endangerment, and trade restrictions. The weights of each species in the multi-species habitat assessment were determined (Table 6). The habitat suitability distributions for the seven species were integrated to generate a multi-species spatial distribution map of suitable habitats (Figure 4). In Figure 4, habitat suitability is represented by a color gradient from blue (low suitability) to red (high suitability). A consistent pattern of high habitat suitability being concentrated in the central part of the study area was observed across multiple species. Overall, the habitat suitability index was higher in the north-central region than in the southern region, and higher in the western region than in the eastern region.

## 4. Discussion

**Figure 5:**
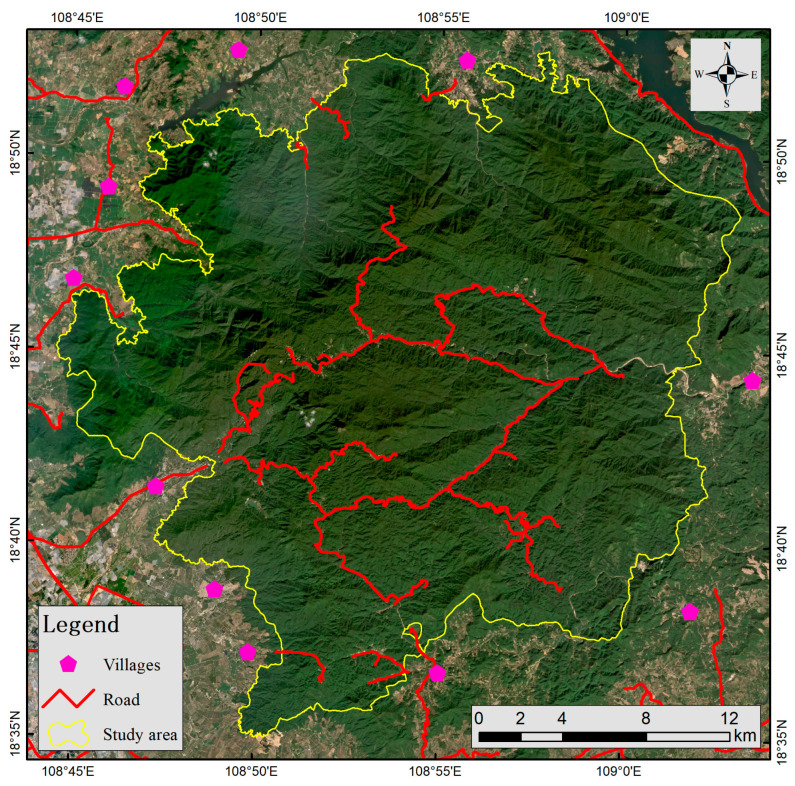
High-resolution remote sensing images and distribution of roads and villages in the study area and its surrounding region.

In the results of the habitat suitability assessment for the seven species, the environmental factors that contributed more than 20% appeared 11 times. Of these, four were distance to roads, three were DEM, two were BIO3, Slope appeared once and EVI appeared once. The average contributions of the seven environmental factors were as follows: Road (21.69%) > DEM (19.05%) > BIO3 (17.06%) > Slope (16.09%) > BIO14 (14.53%) > EVI (12.8%) > Landuse (9.79%). The results demonstrate that distance to roads, or what can be considered as anthropogenic activities, is the most important factor influencing the suitability of wildlife habitats in Jianfengling study area. The avoidance of areas closer to roads by the study species is evident in all suitable habitat distribution maps. This phenomenon is consistent with the findings of several studies [40,41,42]. A comparison of the distribution of roads in and around the study area (Figure 5) and the spatial distribution of multi-species habitat suitability in the study area (Figure 4) reveals a significant reduction in habitat suitability within a certain width along roads. We suggest that this phenomenon results from increased anthropogenic disturbance near roadways, as most wildlife species exhibit pronounced avoidance of human activities. Additionally, specific human interventions—such as hunting and logging—have significant negative effects on both wildlife populations and habitat quality. Notably, our study revealed a distinct pattern among the seven species: all four road-sensitive species (*Muntiacus nigripes*, *Sus scrofa*, *Paradoxurus hermaphroditus*, *Atherurus macrourus*) were ground-dwelling, whereas two of the three less sensitive species (*Macaca mulatta* and *Dremomys pyrrhomerus*) exhibited predominantly arboreal habits. We posit that the dense rainforest canopy likely provides critical visual and physical cover for arboreal species like *Macaca mulatta* [43] and *Dremomys pyrrhomerus* [44], thereby mitigating their perceived vulnerability to anthropogenic disturbances associated with road proximity. It should be noted that the environmental factor data does not fully reflect the impact of other types of anthropogenic activity. For example, the main peak of Jianfengling, situated in the south-western corner of the study area, is a famous tourist attraction, attracting a considerable number of visitors every year. Consequently, it is subject to considerable anthropogenic activities impact. And the final assessment results indicate that this area has a very low habitat suitability.

The DEM is a significant factor influencing wildlife habitat suitability in the study area. The DEM distribution (Figure 1) reveals a topographic pattern with a higher-altitude central region surrounded by lower-altitude areas. This distribution aligns with the habitat preferences of wildlife species, particularly the *Muntiacus nigripes* and *Melogale moschata*, which favor middle and high altitudes, especially within the 500–1000 m range. The higher habitat suitability in these areas can be attributed to several reasons below. Lower-altitude areas, in contrast, exhibit reduced habitat suitability due to their proximity to villages, cultivated land, and other human activities outside the study area (Figure 5), which inevitably affect wildlife activities. This is comparable to the influence of roads. Field surveys show that betel nut, tea, and tropical fruits are cultivated extensively in lower- altitude areas with adequate water sources. Additionally, most villages and townships near Jianfengling study area are inhabited by Li and Miao ethnic groups, some of whom still engage in traditional practices such as mountain walking and hunting. Monitoring data also indicate frequent instances of outsiders entering the mountains for mining and poaching, further exacerbating anthropogenic disturbances. These disturbances are primarily concentrated in lower-altitude regions near villages. In contrast, mid-altitude areas experience minimal human activity, and mid-altitude areas have more stable climatic conditions [45] and richer vegetation diversity [46], providing better living conditions, food sources, and breeding environments [43] for medium and small mammals such as *Muntiacus nigripes* and *Melogale moschata*. However, at elevations above 1000 m, especially beyond 1200 m, vegetation quality declines, and temperatures drop, making the habitat less suitable for most wildlife.

Compared to findings from other habitat assessment studies, where land use strongly influences wildlife habitat selection [47,48], this study found that land use had a relatively smaller impact on the seven species analyzed. This discrepancy may be attributed to the study area being a tropical rainforest with extensive forest cover, where most infrared camera monitoring sites were located within forested areas. Consequently, the model may have lacked sufficient non-forest samples to adequately analyze the relationship between species’ habitat selection and land use types, limiting its ability to evaluate the significant effects of non-forest land types on species distribution. However, the potential influence of land use data—particularly more detailed information, such as tree species distribution—should not be overlooked. Including such data in the model would likely enable more comprehensive analyses and conclusions.

Additional noteworthy phenomena were observed in Jianfengling study area. The *Macaca mulatta* demonstrated a preference for areas with lower slopes and lower EVI values. Combined with findings from related studies [49,50,51], this highlights the diversity of habitat selection in *Macaca mulatta*, a highly adaptable species. Jianfengling study area is classified as a tropical rainforest, where the vegetation quality in regions with relatively low EVI values is also good but has lower canopy density. In the presence of sufficient food resources, low slopes and canopy density provide *Macaca mulatta* with expansive movement space [52], influencing their habitat selection patterns. Among the seven species analyzed in this study, only *Dremomys pyrrhomerus* exhibited a notably high dependency on BIO3 compared to other environmental factors in habitat selection. This could be due to *Dremomys pyrrhomerus* intolerance to high temperatures and their stringent requirements for stable environmental temperatures [53].

Based on the key environmental factors distribution and the findings in Figure 4, which depict the spatial distribution of multi-species habitat suitability, it is recommended that future biodiversity and habitat protection efforts adopt a dual strategy. This strategy should combine routine monitoring in the core areas with targeted monitoring in the surrounding zones. The primary objective is to promptly detect and mitigate the impacts of anthropogenic activities using diverse monitoring techniques. For example, in low-altitude areas and regions near roads, routine inspections should be intensified to identify illegal activities such as farming, hunting, as well as other potential disturbances. Tourism activities within the protected area should be confined to specific zones to minimize their impact on wildlife survival. Regular monitoring, including wildlife surveys and multi-source remote sensing, should be conducted across the entire study area. Additionally, the habitat quality of species in the study area should be periodically reassessed, enabling the adjustment of protection strategies and the implementation of targeted conservation measures based on current conditions.

In terms of methodology, our study utilized ESDM to assess the habitat suitability of seven important species in Jianfengling study area. Compared to the broader application of ESDM in large-scale species habitat suitability prediction and assessment studies [13,14,54,55], we optimized the selection and use of environmental factors and single SDM [56]. Given the relatively small size of our study area, which falls within a mesoscale region, we were able to obtain a greater variety of environmental factors with higher precision, such as multi-type remote sensing factors. This provided a more accurate basis for analyzing species habitat selection. Additionally, in the selection of SDMs, we prioritized those with relatively better average predictive performance for integration, rather than stacking a large number of diverse SDMs. This approach aimed to reduce computational complexity and improve assessment efficiency. Overall, our ESDM framework offers a reliable and efficient methodological choice for habitat suitability prediction and assessment studies in other mesoscale region research.

## 5. Conclusions

In this study, we used the ESDM model to evaluate the spatial distribution of suitable habitats for seven wildlife species in Jianfengling area of Hainan tropical rainforest. This involved analyzing the influence and mechanisms of various environmental factors and calculating the spatial distribution of multi-species habitat suitability. The results revealed that habitat suitability was higher in the central region of Jianfengling area and lower in the low-altitude surrounding zones. Among the seven species analyzed, anthropogenic activities and DEM were the most significant factors influencing habitat selection. Most species preferred mid and high altitude areas (500–1000 m), where the impact of human activities was relatively limited. To protect biodiversity and wildlife habitats, it is recommended that future efforts combine routine monitoring in the central region with targeted monitoring and corrective actions in surrounding areas, focusing particularly on identifying and preventing illegal human activities.

## Figures and Tables

**Table 1:** Overview of information on 7 wild animal species in this study. The symbols “Ⅱ” and “Ⅲ” indicate wildlife that is either nationally priority-protected or falls within the level of protection outlined in the CITES Appendix. Additionally, they represent the Endangered Species Scale: EN: Endangered; VU: Vulnerable; NT: Near Threatened; LC: Least Concern.

Species	No. of Independent Photographs	RAI	National Protected Level	China’s Vertebrate Red List Status	CITES Appendix
*Macaca mulatta*	84	0.198	Ⅱ	LC	Ⅱ
*Muntiacus nigripes*	1027	2.419	Ⅱ	VU	
*Sus scrofa*	1148	2.704		LC	
*Paradoxurus hermaphroditus*	177	0.417	Ⅱ	EN	Ⅲ
*Melogale moschata*	604	1.423		NT	
*Atherurus macrourus*	1838	4.329		LC	
*Dremomys pyrrhomerus*	988	2.327		NT	

**Table 2:** Brief ecological description of 7 wild animal species in this study.

Species	Habitat	Diet and Behavior
*Macaca mulatta*	Exhibits high ecological adaptability, inhabiting a wide range of environments including forests, urban areas, and mountainous regions across South and Southeast Asia.	Omnivorous. Displays complex social structures, forming hierarchical groups.
*Muntiacus nigripes*	Endemic to the dense forests and shrublands of Hainan Island, China.	Herbivorous. Solitary and elusive, with a preference for dense understory vegetation.
*Sus scrofa*	Widely distributed across forests, grasslands, and wetlands in Europe, Asia, and North Africa.	Omnivorous. Highly adaptable and social, forming groups known as sounders.
*Paradoxurus hermaphroditus*	Occupies tropical forests, agricultural plantations, and urban landscapes throughout South and Southeast Asia.	Omnivorous. Nocturnal and arboreal, typically solitary.
*Melogale moschata*	Found in forests, grasslands, and agricultural areas across Southeast Asia.	Omnivorous. Nocturnal and terrestrial, exhibits shy and secretive behavior.
*Atherurus macrourus*	Resides in forests and rocky terrains throughout Southeast Asia.	Herbivorous. Nocturnal and terrestrial, often living in small groups.
*Dremomys pyrrhomerus*	Inhabits montane and subtropical forests in China and Southeast Asia.	Omnivorous. Diurnal and arboreal, adept at climbing and foraging in trees.

**Table 3:** Scale of Conservation Value of Species.

Indicator (Level)	Evaluation Rule	Score
*I_grade_* (National protected level)	I	1
	II	0.5
	III	0.3
	Non-protected	0.1
$I_{endangered}$ (China’s Vertebrate Red List status)	Critically Endangered (CR)	1
	Endangered (EN)	0.8
	Vulnerable (VU)	0.6
	Near Threatened (NT)	0.4
	Least Concern (LC)	0.2
	Not Evaluated (NE)	0.1
$I_{CITES}$ (CITES Appendix)	CITES I	1
	CITES II	0.6
	CITES III	0.3
	Not listed	0.1

**Table 4:** ESDM model prediction accuracy assessment results.

Species	AUC	TSS
*Macaca mulatta*	0.888	0.554
*Muntiacus nigripes*	0.923	0.605
*Sus scrofa*	0.896	0.542
*Paradoxurus hermaphroditus*	0.931	0.648
*Melogale moschata*	0.891	0.540
*Atherurus macrourus*	0.868	0.488
*Dremomys pyrrhomerus*	0.857	0.482

**Table 5:** Information of key environmental factor data.

Type	Factor	Parameter Description	Value Range
Vegetation factor	EVI	High EVI value represents good vegetation quality	−1~1
Topographical factors	DEM	The altitude of each point in the research area	49~1422 m
	Slope	Slope of each point in the research area	0~68.8
Land use/cover factor	Landuse	Land use/cover types at various locations in the research area	-
Meteorological factors	BIO3	Isothermality (BIO2/BIO7×100) BIO2: Mean diurnal temperature range (°C) BIO7: Temperature annual range (°C)	40.29~44.87
	BIO14	Precipitation of the driest month	12.44~25.02 mm
Anthropogenic Activity Factor	Road	The distance from each point in the research area to the nearest road	0~4.93 km

**Table 6:** Conservation value scores and final weighting scores of the seven species’ suitable habitat assessments when participating in the multi-species habitat assessment.

Species	*I_grade_*	$I_{endangered}$	$I_{CITES}$	Weight
*Macaca mulatta*	0.5	0.2	0.6	0.213
*Muntiacus nigripes*	0.5	0.6	0.1	0.197
*Sus scrofa*	0.1	0.2	0.1	0.066
*Paradoxurus hermaphroditus*	0.5	0.8	0.3	0.262
*Melogale moschata*	0.1	0.4	0.1	0.098
*Atherurus macrourus*	0.1	0.2	0.1	0.066
*Dremomys pyrrhomerus*	0.1	0.4	0.1	0.098

## Data Availability

The datasets presented in this article are not readily available because the data are part of an ongoing study. Requests to access the datasets should be directed to yaowt@aircas.ac.cn.
